# Manure combined with chemical fertilizer increases rice productivity by improving soil health, post-anthesis biomass yield, and nitrogen metabolism

**DOI:** 10.1371/journal.pone.0238934

**Published:** 2020-10-07

**Authors:** Anas Iqbal, Liang He, Izhar Ali, Saif Ullah, Ahmad Khan, Aziz Khan, Kashif Akhtar, Shangqin Wei, Quan Zhao, Jing Zhang, Ligeng Jiang

**Affiliations:** 1 Key Laboratory of Crop Cultivation and Farming Systems College of Agriculture, Guangxi University, Nanning, China; 2 Department of Agronomy, the University of Agriculture Peshawar, Peshawar, Pakistan; 3 Institute of Nuclear Agricultural Sciences, College of Agriculture and Biotechnology, Zhejiang University, Hangzhou, China; University of Education Lahore, Pakistan, PAKISTAN

## Abstract

Excessive reliance on chemical fertilizer (CF) in conventional farming is a serious concern owing to its negative effects on soil health, the environment, and crop productivity. Organic manure is an alternative source of fertilizer to reduce the amount of CF usage in agriculture, decrease environmental pollution, and ensure sustainable crop production. This study assessed the integrated effect of poultry manure (PM) and cattle manure (CM) with CF on soil properties, plant physiology, and rice grain yield. Additionally, the difference in pre-and post-anthesis dry matter (DM) and nitrogen (N) accumulation and their relationship with grain yield was also determined. Pot experiments were performed in the early and late growing season at the experimental station of Guangxi University, China, in 2018. A total of six treatments, i.e., T_1_—CF_0_; T_2_−100% CF; T_3_−60% CM + 40% CF; T_4_−30% CM + 70% CF; T_5_−60% PM + 40% CF, and T_6_−30% PM + 70% CF were used in this pot experiment. Results showed that T_6_ enhanced leaf photosynthetic efficiency by 11% and 16%, chlorophyll content by 8% and 11%, panicle number by 12% and 16%, and grain yield by 11% and 15% in the early and late seasons, respectively, compared to T_2_. Similarly1, post-anthesis N and DM accumulation, N uptake, and soil properties (i.e., soil organic carbon, total N, and bulk density) were improved with integrated CF and manure treatments over the sole CF treatments. Interestingly, increases in post-anthesis N uptake and DM production were further supported by enhanced N-metabolizing enzyme activities (i.e., nitrate reductase, glutamine synthetase, and glutamate oxoglutarate aminotransferase during the grain-filling period in combined treatments. In-addition, the linear regression analysis showed that post-anthesis DM (*R*^*2*^ = 0.95) and N (*R*^*2*^ = 0.96) accumulation were highly associated with grain yield of rice. Thus, the combination of 30% N from PM or CM with 70% N from CF (i.e., urea) is a promising option for improvement of soil quality and rice grain yield. Furthermore, our study provides a sustainable nutrient management plan to increase rice yield with high N use efficiency.

## 1. Introduction

Rice (*Oryza sativa* L.) is the main staple food consumed by half of the world’s population and almost 60% of China’s population [1, 2]. Nitrogen (N) is essential for plant growth, so its application influences crop yield by establishing and maintaining photosynthetic and sink capacities [3, 4]. The present farming system is heavily reliant on chemical N fertilizers to achieve higher yields. However, crop yield does not necessarily increase linearly with N fertilizer input [5, 6]. Excessive N fertilization causes significant environmental issues, such as enhanced greenhouse gas emission, groundwater contamination, and surface water eutrophication [7, 8]. Long-term application of N fertilizer has also increased the acidification, degradation, and compaction of arable soils, thereby suppressing plant growth and production [9, 10]. Therefore, this continued reliance on synthetic N fertilizer for cereal crop production is not sustainable. Thus, it is necessary to develop prudent and sustainable management practices that feed the growing population and mitigate environmental costs on a sustainable basis.

On the other hand, organic manure which is derived from animal waste holds great promise, not only to sustain crop production but also to improve soil fertility on a sustainable basis [11, 12]. Manure can substitute mineral fertilizer for increasing crop productivity, carbon sequestration, soil structure, and fertility, as well as reducing environmental pollution [13–15]. Previously, it was well-reported that manure fertilization significantly improved the physicochemical and biological properties of the soil, such as pH, bulk density, enzymatic activity, soil aggregation, soil organic carbon, and both macro- and micro-nutrients [16–18]. Manure fertilization can improve the soil’s physical structure, allowing it to store more water and nutrients, and thus increase crop productivity [19, 20]. Luo et al. [21] reported that organic manure applications with a high N content and low C: N ratio could mineralize enough N to satisfy the demands for plant growth. However, organic fertilizer is quite low in nutrients and its nutrient releasing ability is also slow to meet plant requirements in a short period of time, therefore manure coupled with synthetic fertilizer has been confirmed to be a better approach to improve and sustain soil fertility and crop production than sole application of manure or synthetic fertilizer [22–24].

Cereal grain yield can be obtained from the accumulated dry matter (DM) translocated before and after [25]. Previously, it was documented that 69% of straw N and 84% non-structural carbohydrate accrued pre-anthesis can be translocated to grains, but this depends on sowing conditions and cultivar [26, 27]. Recently, the evidence is now showing that post-anthesis DM production might be a good contributor to cereal yield [25, 28]. However, owing to limited knowledge, further study is required on the role of post-anthesis DM production and its relationship to grain yield. During the grain-filling period, insufficient N uptake in plant leaves and high N translocation from leaves accelerates leaf senescence and reduces the photosynthetic efficiency of the leaf, thus resulting in less assimilation into grain [29]. The N uptake and assimilation obtained by N-metabolizing enzymes pathway, such as nitrate reductase (NR), glutamine synthetase (GS), and glutamate oxoglutarate aminotransferase (GOGAT), increase leaf photosynthetic efficiency and prolong the green period of the leaves and thus ultimately increases post-anthesis DM production [30].

Many studies were conducted on a weight basis rather than the application of manure of a specific N concentration combined with synthetic N fertilizer [31–33]. This has created a research gap in finding out the effects of integrated nutrient management based on specific composition rather than weight. Similarly, information regarding pre-and post-anthesis DM and N accumulation and translocation, and its relationship to rice grain yield is lacking, especially for organic manure coupled with synthetic fertilizer. Therefore, it was assumed for the present study that manure integrated with synthetic fertilizer could improve soil fertility, which in turn will have a positive role in achieving higher biomass and grain yield of rice. The objectives of this study are: (1) to assess the integrated effect of manure and CFs on soil properties; (2) to determine the combined effect of manure and CFs on leaf physiological characteristics, N use efficiency, biomass accumulation, and grain yield; (3) to draw a model to demonstrate DM and N accumulation and their relationship and contribution to rice yield.

## 2. Materials and methods

### 2.1. Experimental location

**Fig 1:**
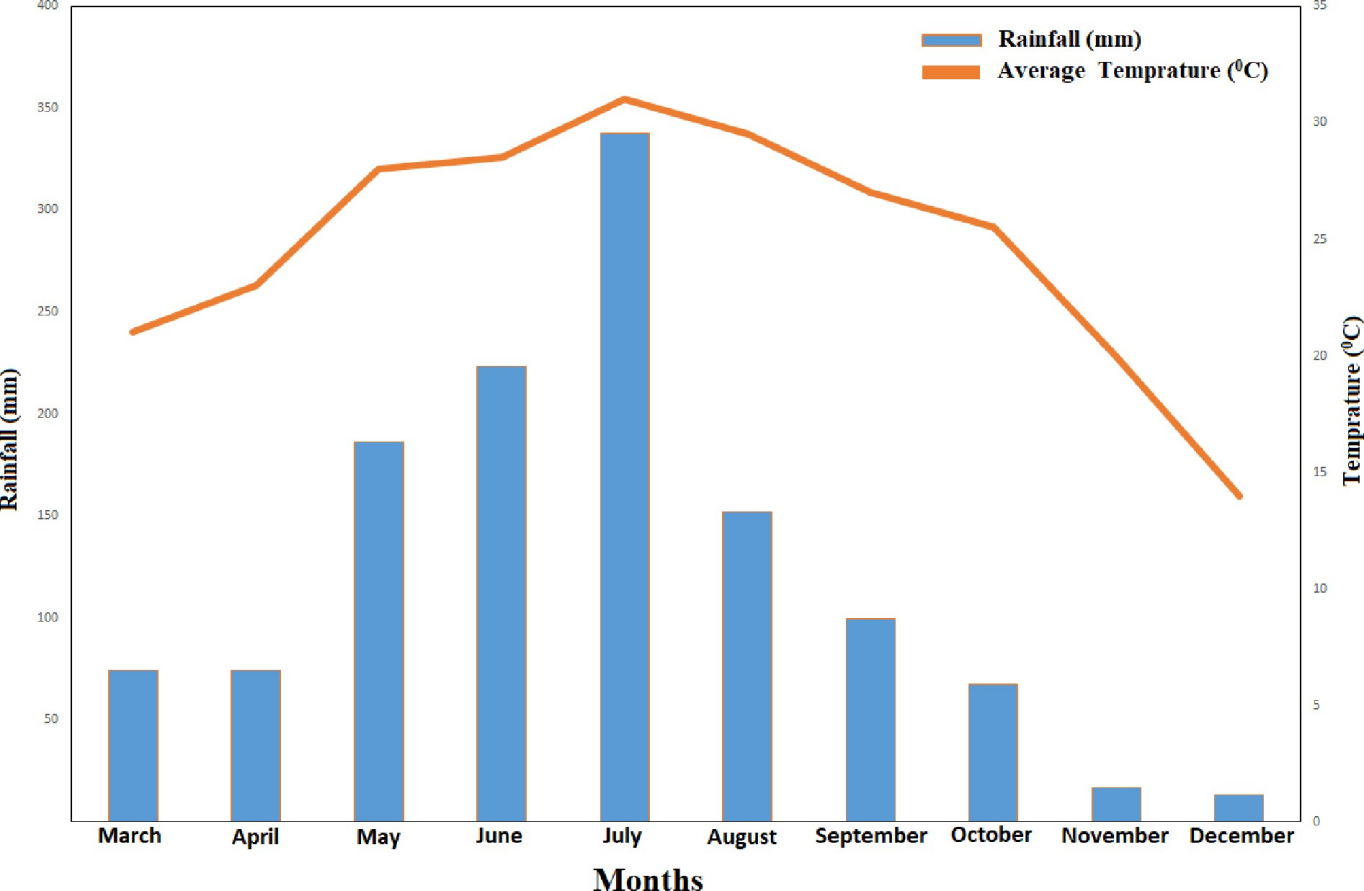
Monthly average temperature and rainfall during the early and late seasons in 2018.

A pot experiment was conducted at the rice experimental research station of Guangxi University, China (22°49′12″ N, 108°19′11″ E; 75 m) during early (March to July) season and was repeated in late (July to November) growing season in 2018. The soil is classified as Ultisols and is acidic in nature with a pH of 5.90. It is low in organic matter (15.2 g kg^-1^), total N (TN) (1.35 g kg^-1^), available N (AN) (130.72 mg kg^-1^), phosphorous (AP) (23.5 mg kg^-1^), and potassium (AK) (232.5 mg kg^-1^), and has a high bulk density (1.36 g cm^-3^). The physicochemical attributes of the soil are shown in Table 1. The climate is classified as subtropical with a monsoon region, with an average annual rainfall of 1080 millimeters. Average maximum and minimum temperature were 33.7°C and 23.2°C, respectively during the early season and 31.6°C and 18.0°C, respectively, in the late season (Fig 1).

**Table 1:** Soil physical and chemical properties before the experimentation.

Properties		Soil	Cattle Manure	Poultry manure
Porosity (%)		40.12	-	-
Moisture content (%)		11.23	-	-
Bulk density (g cm^-3^)		1.38	0.81	0.74
pH (water)		5.95	7.75	7.95
SOC (g kg^-1^)		9.66	146.33	164.22
SOM (g kg^-1^)		16.51	254.63	282.42
Total N (g kg^-1^)		1.34	9.80	12.65
Total P (g kg^-1^)		0.62	10.12	7.32
Total K (g kg^-1^)		-	14.22	9.76
Available N (mg kg^-1^)		130.71	-	-
Available P (mg kg^-1^)		22.21	-	-
Available K (mg kg^-1^)		230.50	-	-
C: N ratio		7.16	14.92	12.98

## 2.2. Treatment structure and experimental setup

The study was conducted under open (natural) field conditions during the dual cropping seasons. Poultry (PM) and cattle (CM) manure were the organic fertilizers and urea was the chemical fertilizer (CF) used in this pot experiment. The experiment consisted of six treatments: T_1_—CF_0_; T_2_−100% CF; T_3_−60% CM + 40% CF; T_4_−30% CM + 70% CF; T_5_−60% PM + 40% CF; and T_6_−30% PM + 70% CF. Plastic pots (20.3 cm width, 29.5 cm length, and 34.1 cm height) were used in this study. A total of fifty-four pots were arranged in a completely randomized design (CRD), with nine replications per treatment, and positioned at a distance of 30 cm between the pots.

The soil for the pots was obtained from the top 20 cm of the experimental research filed. The respective amounts of manure and CFs were added to 1/3 part of the surface soil in the pot. For this purpose, we added 10 out of 14 kg soil to the pots, then added the respective amount of manure to the remaining 4 kg soil and added to the top surface of the soil in the pots. N, phosphorous (P), and potassium (K) were supplied at a ratio of 300:150:300 (kg ha^-1^) and fertilized pots received 1.80 g KCl, 0.90 g P_2_O_5_, and 1.80 g of N from poultry or cattle manure and urea after N estimated. N and KCl were applied in three splits, such as a 60% basal dose, 20% at tillering time, and the remaining 20% at the panicle initiation period, while all P_2_O_5_ was applied before transplantation. Tables 1 and 2 show the chemical composition of CM and PM and the nutrient content and quantity of every pot. The organic manure was obtained from poultry and cattle farms located in the local area. Manure was mixed evenly with soil twenty days before transplantation. The T_1_ (CF_0_) received no N fertilizer, but P_2_O_5_ and KCI fertilizers were added, in equal amounts as those of N-applied pots. Rice cultivar “Zhenguiai” seeds were grown in seedling trays, and 24-day-old seedlings were transplanted into pots, 2 seedlings per hill, and two hills per pot. Standard flood water was provided to a depth of approximately 4 cm from transplantation to physiological maturity. Normal agricultural practices including irrigation, herbicides, and insecticides were performed for every pot.

**Table 2:** Nutrient contents and the amount provided for each treatment and application time.

Treatments	Nitrogen applied from (g pot^-1^)			Application time (g pot^-1^)		
	Total required	Urea	CM/PM	Basal fertilization	Tillering	Panicle initiation
T_1_:	0	0	0	P_2_O_2_: 4.5 KCI: 1.10	KCI: 1.1	
CF_0_						
T_2_:	1.8	3.91	0	Urea: 2.35	Urea: 0.78	Urea: 0.78
100% CF				P_2_O_2_: 4.5 KCl: 1.1	KCI: 1.1	
T_3_:	1.8	1.56	125.8	Urea: 0	Urea: 0.78	Urea: 0.78
60% CM +40% CF				CM: 125.8		
				P_2_O_2_: 4.5 KCI: 1.1	KCI: 1.1	
T_4_:	1.8	2.73	62.9	Urea:1.17 CM: 62.9 P_2_O_2_: 4.5 KCI: 1.1	Urea: 0.78	Urea: 0.78
30% CM +70% CF						
					KCI: 1.1	
T_5_:	1.8	1.56	108.2	Urea: 0	Urea: 0.78	Urea: 0.78
60% PM + 40%CF				PM: 108.2		
				P_2_O_2_: 4.5 KCI: 1.1	KCI: 1.1	
T_6_:	1.8	2.73	54.1	Urea: 1.17PM: 54.1	Urea: 0.78	Urea: 0.78
30% PM +70%CF						
				P_2_O_2_: 4.5	KCI: 1.1	
				KCI: 1.1		

Note: N—nitrogen, CF_0_—control, CF—chemical fertilizer (urea), CM—cattle manure, PM—poultry manure, P_2_O_2_—superphosphate, KCI—potassium chloride.

### 2.3. Sampling and measurements

#### 2.3.1. Manure and soil sampling and analysis

Initial soil and organic manure sub-samples were taken randomly, air-dried, and passed through a 2 mm sieve. Similarly, three replicated soil samples were collected from up to 20 cm depth for each treatment after rice harvest during both the early and late growing seasons to determine the changes in the soil’s physical and chemical properties. The soil samples were air-dried at room temperature and divided into two sub-samples; one was used for immediate analysis and the other was stored at 4°C for further analysis. The basic soil properties are shown in Table 1.

Soil organic carbon (SOC) was measured using the oxidation method. Subsoil samples (0.5 g) were boiled with 5mL of 1 M K_2_ Cr_2_O_7_-H_2_SO_4_ and 5 mL of concentrated H_2_SO_4_ at 170°C for 6 min, cooled, and titrated against 0.4N FeSO_4_ according to the method of Bao [34]. For total soil N content, 200 mg soil was digested using salicylic acid, sulfuric acid hydrogen peroxide as per the procedure of Ohyama et al. [35], and then total N was analyzed by the micro-Kjeldahl method [36]. Phosphorous was determined by the procedure of Murphy, [37]. Soil available N (AN) was estimated using the method defined by Kostechkas [38] and Dorich [39]. Total potassium was measured by preparing a standard stock solution of KCl in distilled water and the measurement was taken at wavelength 7665 R by anatomic absorption spectrophotometer (Hitachi-Z-5300, Japan). The soil bulk density (BD) was determined by the core method, according to Grossman, [40].

#### 2.3.2. Photosynthetic rate and chlorophyll content

Flag leaf photosynthetic (*Pn*) and chlorophyll content (Chl), i.e., Chla and Chlb were estimated during the reproductive period. The photosynthetic rate was determined from the flag leaf that fully expanded in all pots using a photosynthesis machine (Li-COR-6400, United States). Data were collected on a sunny day from 10:00 a.m. to 12:30 p.m. with the following conditions; light intensity 1200 μmol m^-2^s^-1^, air humidity 75%, CO_2_ 370 μmol mol^−1^, and leaf temperature 28°C.

For leaf chlorophyll content measurement, the first 1.0 g of leaf tissue was thinly sliced and added to a volumetric flask containing 10 mL of 80% acetone solution, according to the method of Porra et al. [41], and then placed in the dark for 24 h. The absorbance of the extracted solution was calculated at 663 and 645 nm by spectrophotometer UV (Tecan-infinite M200, Switzerland). The equation recommended by Arnon [38], was used to quantify chlorophyll a and b content:

$$C_{\left(Chla\right)}=12.71D_{663}-2.59\;D_{645}$$ (1)

$$C_{\left(Chlb\right)}=22.88\;D_{645}-4.67\;D_{663}$$ (2)

Where, C_(Chla)_ and C_(Chlb)_ are the content of Chla or Chlb, respectively; D_663_ and D_645_ are the absorbances at 663 and 645 nm, respectively.

#### 2.3.3. N metabolism enzyme activities

Three flag leaves from each pot were taken during the grain-filling time, instantly frozen in liquid N, and placed in −80°C to assess the N-metabolizing enzyme activities, such as GS, NR, and GOGAT. The NR activity was measured using the procedure recommended by Li, [42]. The activity of NR present in μmol NO_2_ per gram fresh weight h^-1^, indicating that one mol NO_2_ formed in one hour from1g of fresh leaf weight at 25°C. The activity of GS was measured with the procedure of Lee et al. [43]. One unit of GS enzyme activity was equivalent to the quantity of enzyme catalyzing the production of one μmol of glutamyl hydroxamate per min at 37°C. The procedure of Sing and Srivastava [44] was used to calculate the GOGAT activity, where 1 unit is defined as decreasing one μmol nicotinamide adenine dinucleotide in the reaction mixture per minute at 30°C.

#### 2.3.4. Dry matter, N accumulation, and translocation

At anthesis and physiological maturity, three replicates for each treatment were used for the determination of DM and N accumulation. Rice plants were separated into stems + leaf sheaths, leaves, and panicles, then dried in oven at 85°C until reaching a stable weight. The samples were ground to make powder, and total nitrogen (TN) was estimated according to Jackson [36], using the micro-Kjeldhal process. Post-anthesis DM and N accumulation are considered to be the difference between anthesis and maturity of the aboveground accumulation, using the method proposed by Pal et al. [25]. This assumes that the entire DM and nitrogen losses from the plant’s vegetative organs have been transferred to the grains. The nitrogen transaction (NT) and dry matter translocation (DMT) in the grain-filling phase were measured according to the equations suggested by Papakosta and Gagianas [45]:

$$DMT=DMa-(DM_{leaf,m}+DM_{stem,m}+DMchaff,m)$$ (3)

$$NT=NTa-(NT_{leaf,m}+NT_{stem,m}+NTchaff,m)$$ (4)

where DMa is the total DM accumulation of aboveground plant at anthesis stage and DM_stem,m_, DM_leaf,m_, and DMchaff,m are the DM of leaves, stems, and chaff, respectively, at maturity stage. NTa is the total N accumulation of the aboveground plant at the anthesis stage, and NT stem,m, NT_leaf,m,_ and NTchaff,m are the total N accumulation of stems, leaves, and chaff, respectively, at physiological maturity. The DM translocation (DMTE) and N translocation (NTE) efficiency were calculated according to the equations:

DMTE=DMT/DMa×100% (5)

NTE=NT/NTa×100% (6)

The contribution of pre-anthesis DM remobilization to grain (DMRG) and N assimilation to grain (NRG) were estimated as:

DMRG=DMT/DMgn×100% (7)

NRG=NT/Ngn×100% (8)

DMgn and Ngn are the DM and N content of grain at physiological maturity, respectively. In-addition, the N indexes, including agronomic N use efficiency (AE_N_), recovery efficiency (RE_N_), partial factor productivity (PFP_N_), and internal N use efficiency (IE_N_) were estimated using the following equations:

$$PFP_{N}=G_{N}/F_{N}$$ (9)

$$IE_{N}=G_{N}/T_{N}$$ (10)

$$AE_{N}=(G_{N}-G_{0})/F_{N}$$ (11)

$$RE_{N}=(T_{N}-T_{0})/F_{N}$$ (12)

where T_0_ and T_N_ are total N uptake in the plants without N and N-treated pots, G_0_ and G_N_ are the yields in the subsequent pots, and F_N_ is the amount of N supplied.

#### 2.3.5. Yield and yield components

The crop was harvested when almost all heads showed complete loss of green color. Both hills were collected from each pot to assess the panicle number, spikelets per panicle, grain-filling rate, 1000 grain weight, and grain yield at maturity.

#### 2.3.6. Statistical analysis

The recorded data on soil properties, plant physiological attributes, grain yield, and yield components were analyzed according to the ANOVA techniques relevant to CR design using Statistics 8.1 analytical software. The collected data were first checked for normal test. Data in percentage were arcsine transformed to normalize the variables before analysis. The analysis was conducted combined over the seasons, to detect differences between seasons in addition to the fertilizer treatments. The experiment consisted of a single factor (i.e., fertilizer treatments were a fixed factor); however, it was repeated for the following season, thus season was also considered as a repetitive measured factor and also a fixed effect. Similarly, the interaction between fertilizer treatments and the season was taken as a fixed effect. However, the interaction of season and treatment with replications was taken as a random effect. The means were separated using Tukey’s test at *p* < 0.05. Linear regression analysis was performed to evaluate the relationship between *Pn*, panicle number, number of spikelets per panicle, and DM and N accumulation.

## 3. Results

### 3.1. Soil qualitative attributes

The combined fertilization of chemical fertilizer with poultry or cattle fertilizer had a positive influence on soil health. Soil quality traits, such as BD, SOC, TN, and AN were considerably different among the treatments and seasons (Table 3). The significance of ANOVA is presented in Table 3. Soil properties were significantly affected by different treatment and season, whereas ST did not affect soil properties. The integrated treatments significantly improved the soil quality attributes across the seasons. Compared to T_2_, T_3_ significantly increased SOC by 17% and 32%, TN by 19% and 31%, AN by 12% and 21% during the early and late seasons, respectively. Similarly, the T_3_ decreased the soil BD by 7 and 12% during the early and late seasons, respectively, compared to T_2_. However, T_5_ was statistically non-significant (*p* < 0.05) with T_3_. Similarly_,_ the T_6_ and T_4_ also improved the soil quality attributes more significantly than T_2_.

**Table 3:** Changes in soil physicochemical properties under combined organic and inorganic N fertilization.

Treatments	BD	SOC	TN	AN
	(g cm^-3^)	(g kg^-1^)	(gkg^-1^)	(mgkg^-1^)
Early season				
T_1_	1.37 a	9.60c	1.31c	131.1c
T_2_	1.38 a	9.65c	1.35c	134.5c
T_3_	1.24 c	11.28a	1.61a	152.4a
T_4_	1.31 b	10.40b	1.46b	146.5b
T_5_	1.25c	11.30a	1.62a	153.2a
T_6_	1.32b	10.44b	1.46b	148.5b
Average	1.31a	10.43b	1.46b	144.36b
Late season				
T_1_	1.37 a	9.61c	1.29c	128.3c
T_2_	1.37 a	9.66c	1.33c	136.5c
T_3_	1.19d	12.76a	1.83a	171.5a
T_4_	1.26 b	11.96b	1.69b	158.5b
T_5_	1.20 c	12.80a	1.85a	172.2a
T_6_	1.26 b	12.00b	1.68b	159.2b
Average	1.27b	11.47a	1.61a	154.366a
ANOVA				
Treatments	**	**	**	**
Season	*	*	*	*
S × T	Ns	ns	ns	ns

Note: T_1_-CF_0_, T_2_-100% CF, T_3_-60% CM + 40% CF, T_4_-30% CM + 70% CF, T_5_-60% PM+ 40% CF, T_6_-30% PM + 70% CF, CF—Chemical fertilizer, CM—cattle manure, PM—poultry manure, BD—Bulk density, SOC—Soil organic carbon, TN—Total nitrogen, AN—Available nitrogen. Values followed by the same letters, within column are statically same at *p* ≤ 0.05. The mean comparison were made using tukey tests for treatments mean in both seasons and the lettering was done on basis of Tuky HSD test at 5% using simple effect. ^ns^ = non-significant

*, ** = significant at 5% and ** at 1%, respectively.

## 3.2. Flag leaf net photosynthetic rate and Chl content

**Fig 2:**
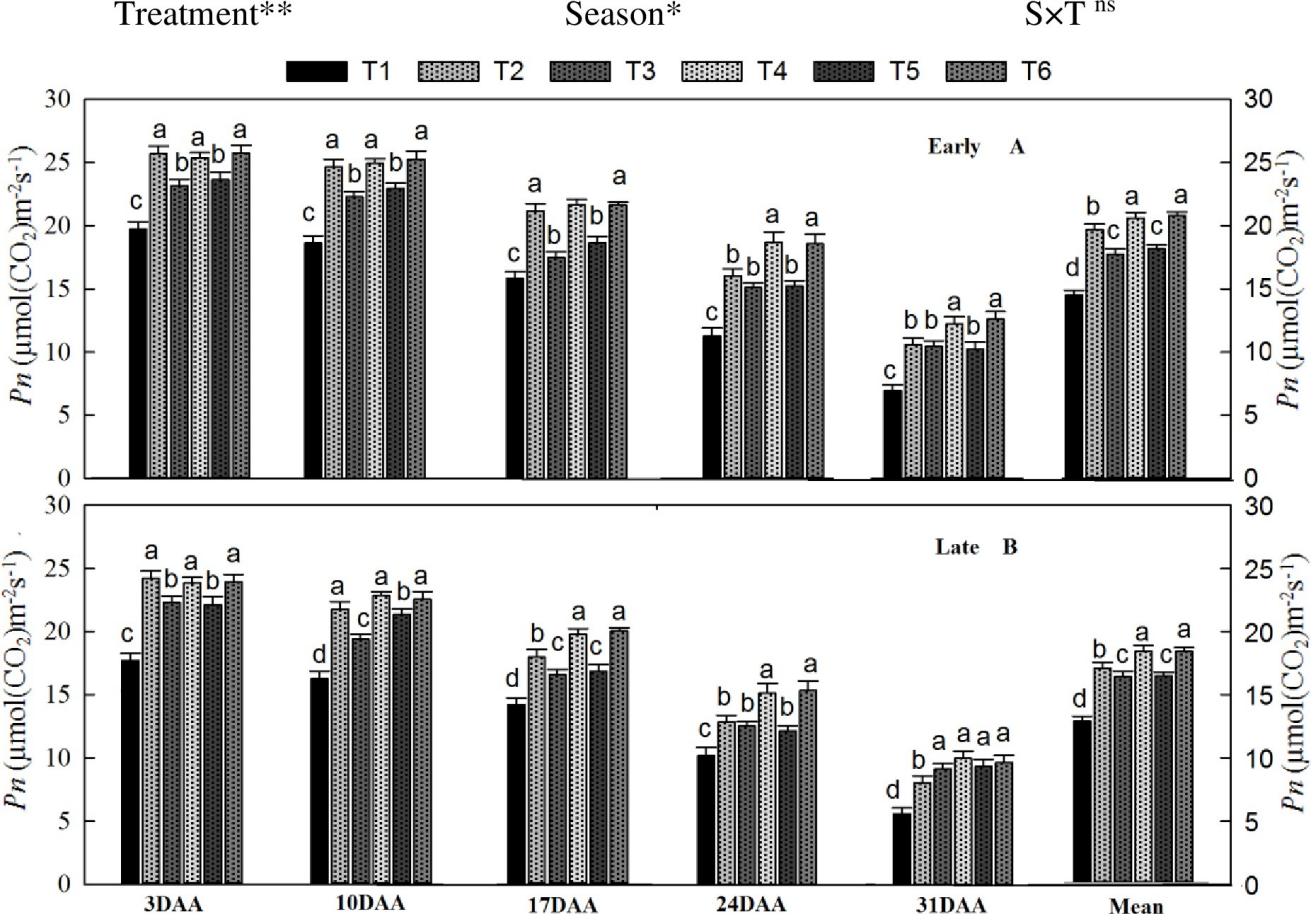
Changes in net photosynthetic rate (3, 10, 17, 24, and 31 days after anthesis (DAA)) during grain-filling period during in the early (A) and late growing season (B) under combined organic and inorganic N fertilizer application. Vertical bars represent the standard error of the mean. Different letters above the column indicate significance at the (*P* < 0.05). Note. *Pn*—net photosynthetic rate, CF—chemical fertilizer, CM—cattle manure, PM—poultry manure. The treatment coding details already showed in the above figure. The mean comparison was made using Tukey tests for treatments mean in both seasons and the lettering was done on basis of Tuky HSD test at 5% using simple effect. ns = non-significant; *, ** = significant at 5% and ** at 1%, respectively.

**Fig 3:**
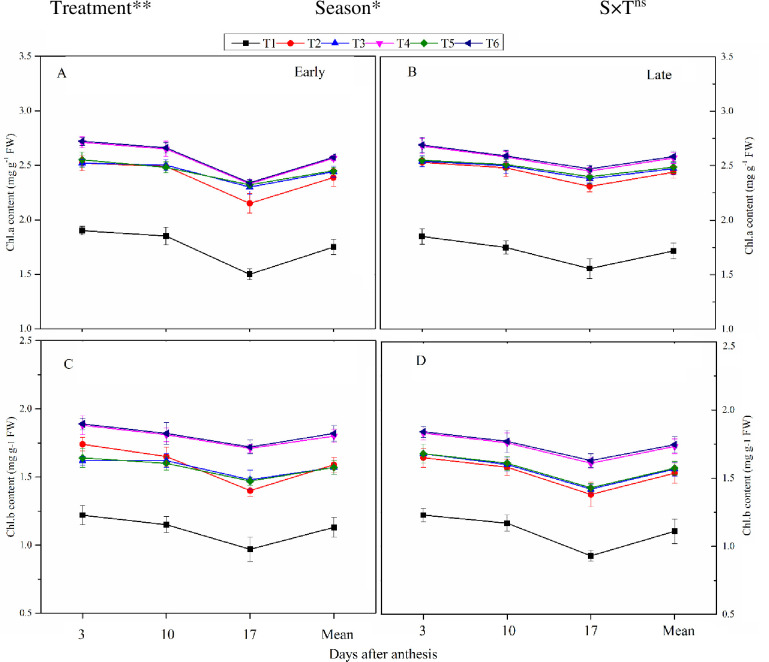
Changes in flag leaf chlorophyll content a and b (3, 10, and 17 days after anthesis—DAA) during grain-filling at the early (A, C) and late (B, D) season under the combined organic and inorganic N fertilization. Vertical bars represent the standard error of means. Note. Chl.a—Chlorophyll a, Chl.b—Chlorophyll b, FW—Fresh leaf weight. The treatment coding details already showed in the above figure. The mean comparison was made using Tukey tests for treatments mean in both seasons and the lettering was done on basis of Tuky HSD test at 5% using simple effect. ns = non-significant; *, ** = significant at 5% and ** at 1%, respectively.

Flag leaf net photosynthetic rate (*Pn*), Chla, and Chlb during the grain-filling period, significantly affected by different organic and inorganic N fertilizer treatments and season, while the interaction of treatments and seasons was not non-significant (Figs 2 and 3). The *Pn* showed a declining trend with days after anthesis (DAA), as shown in Fig 2A and 2B. Flag leaf *Pn* was higher during the early grain-filling period and then decreased with DAA. Across the grain-filling period, T_6_ increased *Pn* by 12% and 13% during the early and late seasons, respectively, compared to T_2_. However, T_4_ was noted non-significant with T_6_. Similarly, T_3_ and T_5_ also enhanced *Pn* compared with T_2_. The lowest *Pn* was recorded in non-N treated pots during both seasons.

The flag leaf chlorophyll content, Chla and Chlb, also exhibited a declining trend with DAA (Fig 3). Flag leaf Chla and Chlb were considerably different among the treatments and seasons. Across the grain-filling period, T_6_ increased Chla by 12% and 14% and Chlb by 14% and 16% during the early and late growing seasons, respectively, compared to T_2_. However, T_4_ was observed statistically similar to T_6_. Likewise, T_3_ and T_5_ also improved the Chl content compared to T_2_ and T_1_. The lowest flag leaf Chl content was recorded in T_1_ during both seasons.

### 3.3. The activity of N-metabolizing enzymes

**Fig 4:**
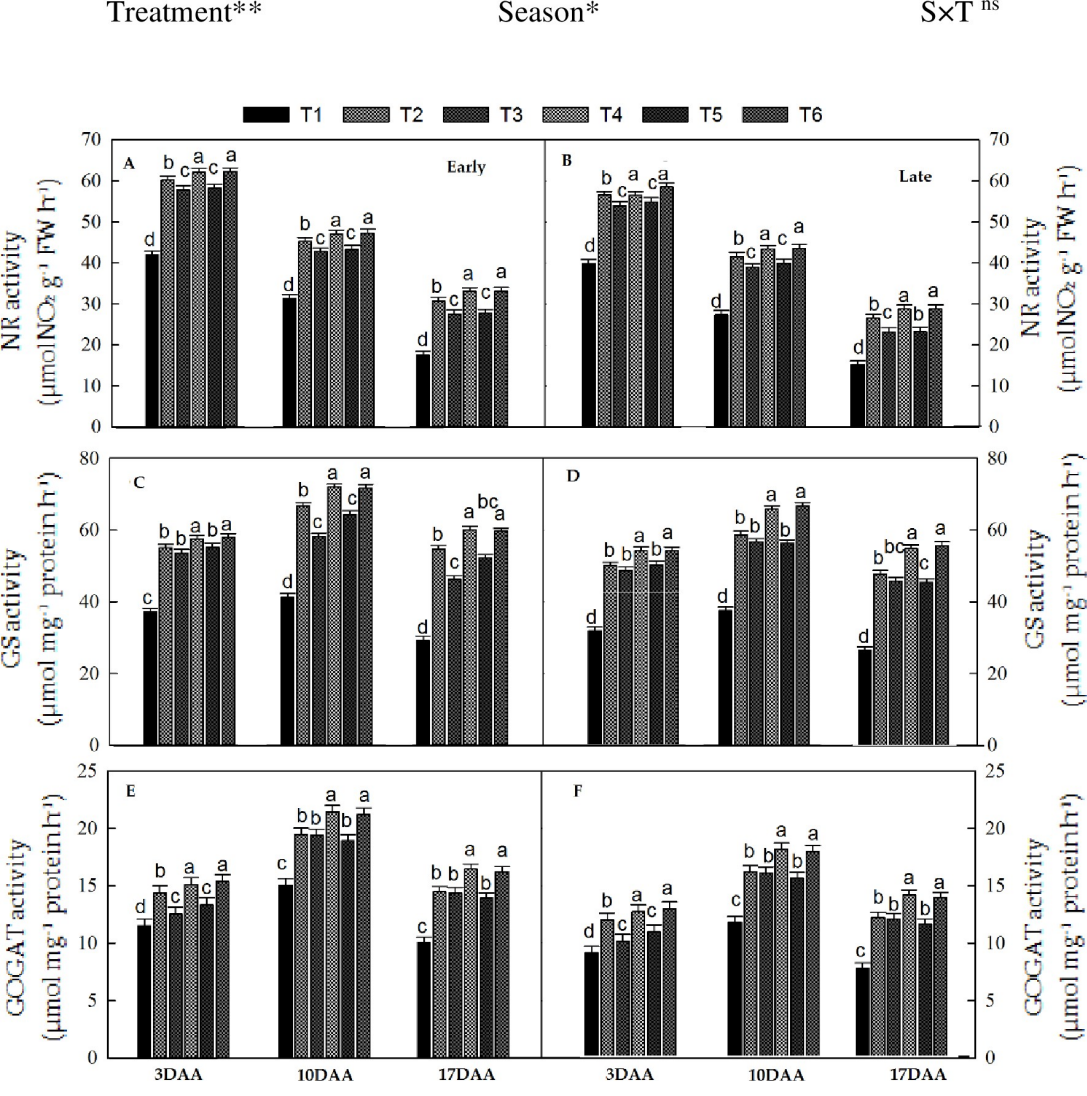
Changes in N metabolism enzymes activities (3, 10, and 17 days after anthesis (DAA)) during grain filling period, NR, GS and GOGAT at early season (A-C-E) and late season (B-D-F) in response to combined organic and inorganic N fertilizer application. The vertical bar represents the standard error of the mean. Different letters above the column indicate statistical significance at the (*P* < 0.05). Note: NR—nitrate reductase, GS—glutamine synthetase, GOGAT—glutamine 2-oxoglutarate aminotransferase. The treatment coding details already showed in the above figure. The mean comparison was made using Tukey tests for treatments mean in both seasons and the lettering was done on basis of Tuky HSD test at 5% using simple effect. ns = non-significant; *, ** = significant at 5% and ** at 1%, respectively.

The activity of N-metabolizing enzymes, such as NR, GS, and GOGAT during grain-filling were significantly influenced by organic and inorganic N fertilizer treatments and season, whereas the interaction of treatments and seasons were not non-significant (Fig 4). The activity of N-metabolizing enzymes for all treatments showed the same behavior during both seasons. The NR activity showed a decreasing trend during the grain-filling phase, higher at 3 DAA, then slowly decreased and was lowest at 17 DAA (Fig 4A and 4B). Averaged across the grain-filling period, NR activity in T_6_ was 10% and 12% higher during the early and late seasons, respectively, compared to T_2_. However, T_4_ was observed non-significant with T_6_. Similarly, T_3_ and T_5_ also improved NR activity, while the lowest values were observed in T_1_.

In-contrast, GOGAT, and GS enzyme activity displayed an upward and downward pattern during the grain-filling period. The highest GS and GOGAT peak activity occurred at 10 DAA and then decreased (Fig 4C–4F). Averaged across the DAA, the values of GS and GOGAT for T_6_, were 10% and 11% (early season) and 12% and 13% (late season) higher than those of T_2_, respectively. However, no significant difference was noted between T_6_ and T_4_. Likewise, T_5_ and T_3_ also had enhanced the GS and GOGAT activity compared to T_2_ and T_1._

### 3.4. Nitrogen indexes

**Fig 5:**
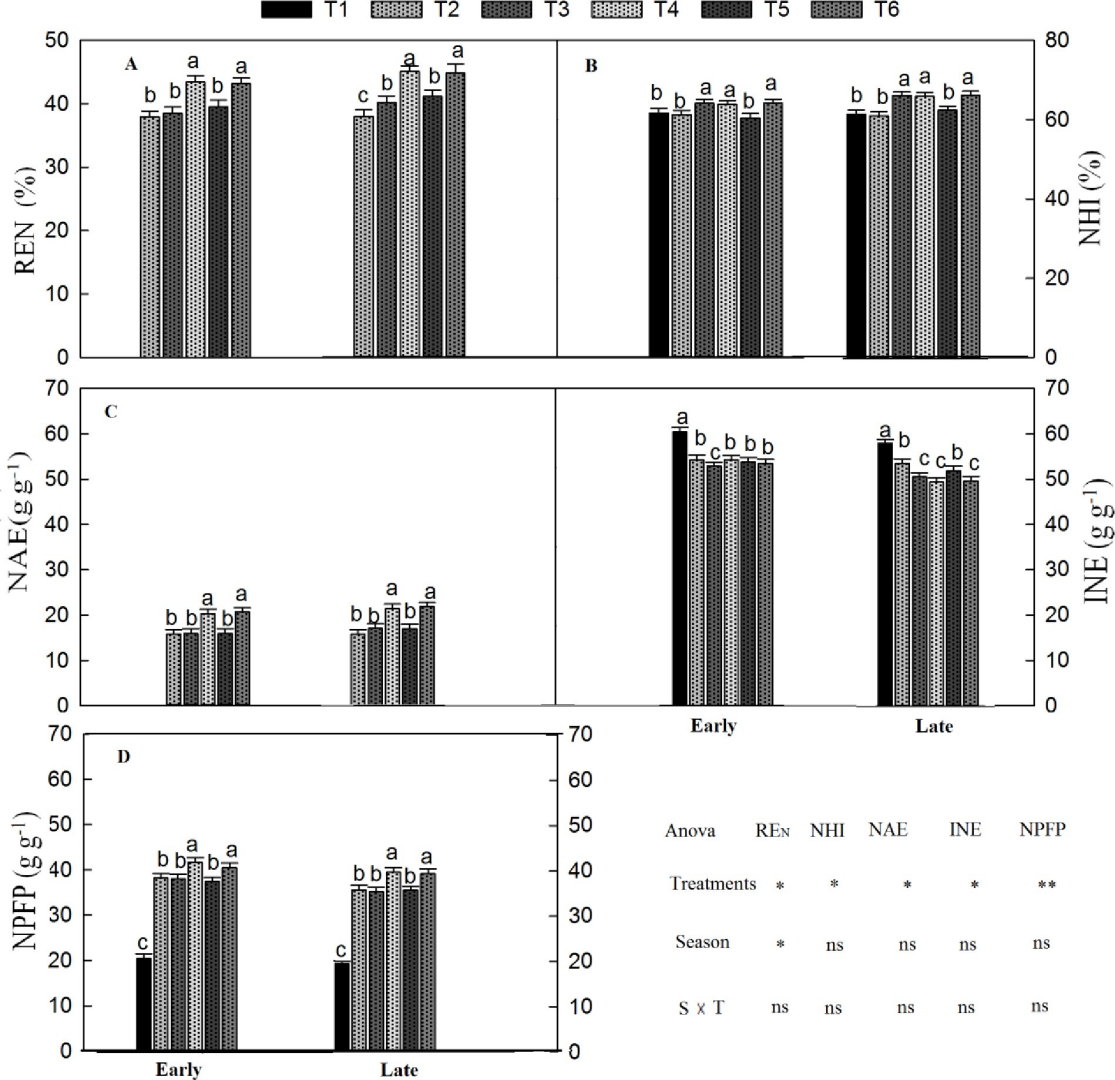
Comparison of N uses efficiencies under combined organic and chemical N fertilizer application. N recovery efficiency (REN) (A), N harvest index (NHI) (B), N agronomic efficiency (NAE) (C), internal N efficiency(INE) (D), and N partial factor productivity (NPFP) (D) early and late season. The vertical bar represents the standard error of the mean. Different letters above the column indicate statistical significance at *P* < 0.05. The treatment coding details already showed in the above figure. The mean comparison was made using Tukey tests for treatments mean in both seasons and the lettering was done on basis of Tuky HSD test at 5% using simple effect. ns = non-significant; *, ** = significant at 5% and ** at 1%, respectively.

**Fig 6:**
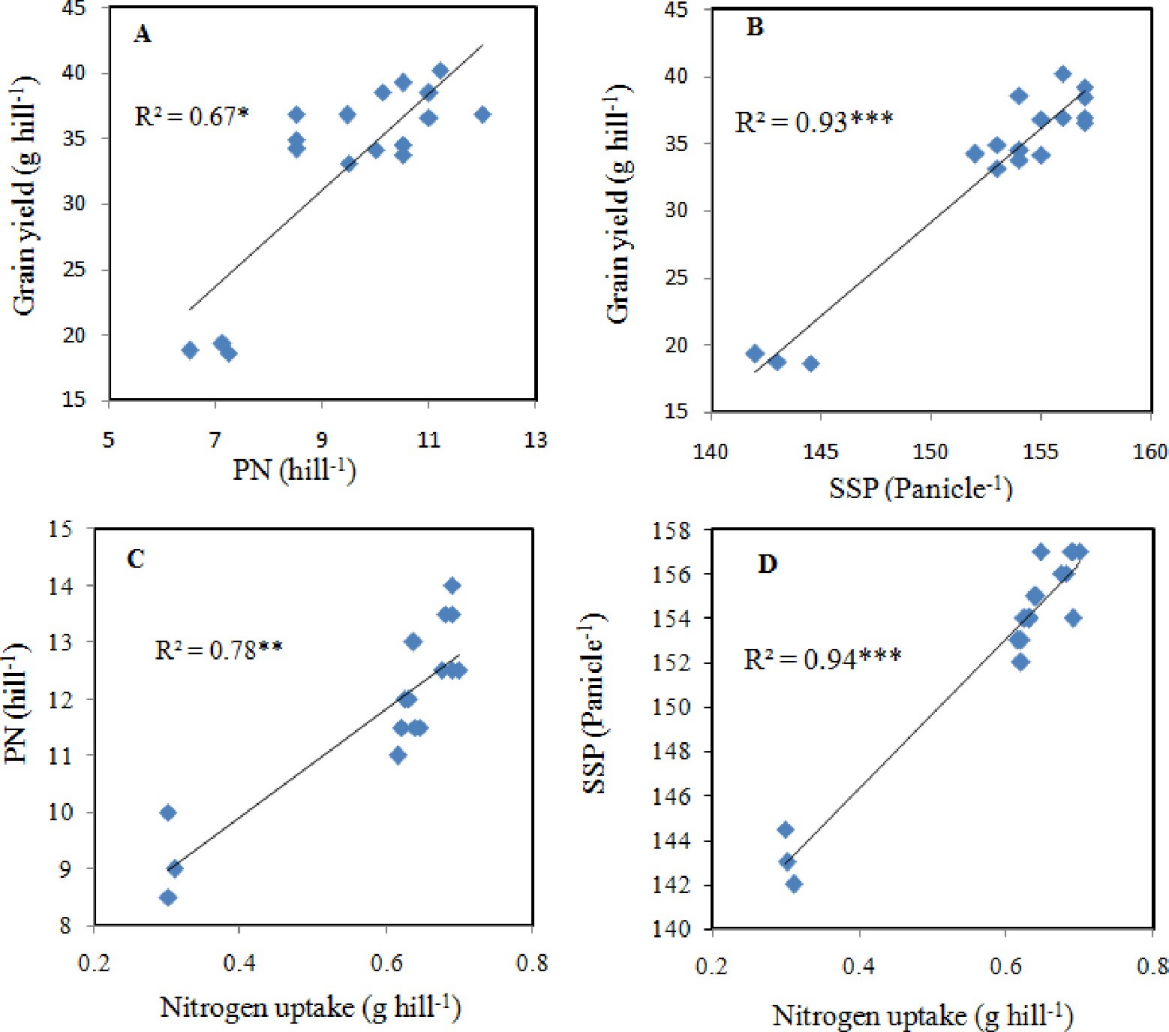
Linear relationship of grain yield with panicle number [PN, (A)] and spikelet number panicle -1 [SSP, (B) and PN (C) and SSP (D)] with N uptake *** *P* < 0.001.

The N indexes (i.e., NHI, NAE, IEN, and NPFP) were significantly influenced by combined organic and inorganic N fertilizer treatments and season, whereas the interaction of treatments and seasons were not non-significant (Fig 5). Moreover, the N recovery efficiency was also significantly affected by different seasons. The N harvest index (NHI) of T_6_ was 65% and 68% in the early and late season, respectively, the highest of all other treatments as shown in Fig 6B. However, T_6_ was statistically similar to T_4_. The lowest NHI was observed in T_2_ which resulted in 63% and 61% NHI in the early and late seasons, respectively. As shown in Fig 5C and 5D, T_2_ had agronomic N efficiency (AE_N_) of 17.48 and 15.67 g g^-1^ in the early season and late season, respectively. Relative to T_2_, treatment T_6_ significantly increased AE_N_ by 13% and 16%, respectively, in the early and late seasons.

The internal N efficiency (IE_N_) T_1_ had maximum IE_N_ in the early (61 g g^-1^N) and late (63 g g^-1^N) seasons, compared to the fertilized treatments (Fig 5C and 5D). In the early season, the IE_N_ was not different among the fertilized treatments. However, T_6_, in the late growing season, had significantly lower IE_N_ (9%) than T_2_. However, T_3_ and T_4_ were non-significant with T_6_. The plants in T_2_ had N partial factor productivity (PFP_N_) of 41.2 and 38.5 g g^-1^ N in the early and late seasons, respectively (Fig 5C and 5D). Compared to T_2_, T_4_ significantly increased PFP_N_ by 8.4% and 13.2% in the early and late seasons, respectively. Similarly, T_3_ and T_5_ also improved PFP_N_ compared to T_1_. Integrated organic and chemical N fertilized pots had significantly higher N uptake and N use efficiencies in the early season and late season, compared to CF (Fig 5). The RE_N_ varied considerably between the treatments in the early and late season (Fig 5A). The treatment T_6_ had higher RE_N_ rates of 43% and 45% during the early and late season, respectively. Similarly, T_5_ and T_3_ also improved the RE_N_ over CF. The lowest RE_N_ values were recorded in the CF treatment (T_2_) and had 38% and 37% during early and late seasons, respectively.

### 3.5. Dry matter accumulation and translocation

In the present study, plant DM accumulation and translocation were significantly affected by combined organic and inorganic N fertilizer treatments and season, whereas the interaction of treatments and seasons was not non-significant (Table 4). The treatments T_6_ and T_4_ had the greatest DM production, followed by treatments T_2_, T_3,_ and T_5_, while minimum DM accumulation was recorded in T_1_ (Table 4). At maturity, for T_2_ the total DM was 67.5 and 60.8 g hill^-1^ during the early season and late season, respectively. Compared to T_2_, the treatment T_6_ considerably increased DM by 8% and 12% during the early and late season, respectively. However, T_6_ was statistically (*p* < 0.05) comparable with T_4_. In-addition, T_3,_ and T_5_ also improved DM production over T_1_ and were statistically similar to T_2_. In-addition, the integrated use of organic and inorganic fertilizers significantly enhanced post-anthesis DM production (Table 3). Relative to T_2_, treatment T_6_ enhanced DM after anthesis and was 30.2 and 23.8 g hill^-1^ in early season and late season, respectively, which was 9.4% and 17.8% higher than T_2_, in the early and late seasons, respectively.

**Table 4:** Dry matter accumulation at anthesis, physiological maturity, and post-anthesis accumulation, dry matter translocation, dry matter translocation efficiency, and contribution to grain yield under different organic and inorganic N fertilizer application.

				DMT	DMTE	CDMRG
		DM (ghill^-1^)				
Treatment	Ant	Mat	Pos-A	(g hill^-1^)	(%)	(%)
Early		41.6c	12.9c			
T_1_	28.7c			6.1c	20.8a	31.7a
T_2_	39.9a	67.5b	27.6b	7.4a	18.2b	20.5b
T_3_	36.8b	66.0b	29.2a	6.6bc	17.8b	19.4b
T_4_	40.6a	71.0a	29.8a	7.4a	18.0b	19.3b
T_5_	39.4b	66.7b	27.4b	6.8bc	17.3b	19.8b
T_6_	41.1a	72.6a	30.2a	7.5a	18.2b	20.2b
Average	37.9a	64.2a	26.a	7.0b	18.4b	21.8b
Late		39.3c	10.9c			
T_1_	28.4d			9.1bc	31.5a	51.6a
T_2_	41.4ab	60.8b	20.2b	11.7a	28.4b	37.5b
T_3_	38.3c	60.1b	21.8ab	10.1b	26.3b	31.6b
T_4_	42.1a	65.6a	23.7a	12.1a	28.8b	34.1b
T_5_	40.7b	62.1b	22.7a	9.5b	25.3b	29.5b
T_6_	42.6a	66.6a	23.8a	12.1a	26.7b	33.1b
Average	38.2a	61.0b	22.4b	10.8a	27.8a	36.2a
ANOVA						
Treatments	**	**	**	*	*	**
Seasons	ns	*	*	*	*	*
S × T	ns	ns	ns	ns	ns	ns

Note: T_1_-CF_0_, T_2_-100% CF, T_3_-60% CM + 40% CF, T_4_-30% CM + 70% CF, T_5_-60% PM+ 40% CF, T6-30% PM + 70% CF, CF—chemical fertilizer, CM—cattle manure, PM—poultry manure, Ant-anthesis, Mat—physiological maturity, Pos-A—post anthesis accumulation, DMT—dry matter translocation, DMTE—dry matter translocation efficiency, CDMRG—contribution to grain yield (CDMRG), DM—dry matter. The mean comparison in columns were made using tukey tests for treatments mean in both seasons and the lettering was done on basis of Tuky HSD test at 5% using simple effect. ^ns^ = non-significant

*, ** = significant at 5% and ** at 1%, respectively.

Post-anthesis dry matter translocation (DMT) in T_1_ was the lowest among all treatments during both seasons, as shown in Table 4. The DMT in N applied treatments, i.e., T_2_, T_4,_ and T_6_ were found statistically comparable, followed by treatments T_3_ and T_5_. Nevertheless, the N applied regimes had considerably lowered dry matter translocation efficiency (DMTE), compared to T_1_. No significant variation was observed among T_6_, T_5_, T_4_, T_3_, and T_2_ for DMTE across the seasons. Similarly, the highest values of post-anthesis DMT contributed to grain yield were 31.7% and 51.6% in the early season and late season, respectively, in T_1_. All the N fertilizer applied treatments, such as T_2_, T_3_, T_4_, T_5,_ and T_4_ were noted to be comparable.

## 3.6. N accumulation and translocation

The organic manure applied with CF significantly increased N recovery at both anthesis and maturity, relative to CF across both seasons (Table 5). Further, the ANOVA showed N accumulation and translocation were considerably influenced by different manure and synthetic N treatments and season, while the interaction of treatments and seasons was not non-significant as shown in Table 5. The T_6_ had maximums of 0.44 and 0.42 (g hill^-1^) N accumulation at anthesis, and 0.66 and 0.68 (g hill^-1^) N accumulation at maturity. Compared to CF only, the combined treatments T_4_ and T_6_ increased N accumulation by 5.2% and 9.7% at maturity, respectively, during the early and late season. However, T_4_ was recorded statistically comparable with T_6_. Further, T_3_ and T_5_ also increased N accumulation over T_1_. Maximum post-anthesis N uptake was 0.25 and 0.27 g hill^-1^ in T_6_ in the early and late season, respectively. However, T_4_ was found non-significant compared to T_6_. Accordingly, after anthesis plant N accumulation was in the order of T_4_, T_6_ > T_2_, T_5_, T_3_ > T_1_.

**Table 5:** Nitrogen uptake at anthesis, physiological maturity, and post-anthesis accumulation, N translocation, N translocation efficiency, and contribution to grain N under different organic and inorganic N fertilizer treatment.

		NA (g hill^-1^)		NT	NTE	CNRG
Treatment	Ant	Mat	Pos-A	(g hill^-1^)	(%)	(%)
Early		0.37c	0.14c			
T_1_	0.23c			0.15c	64.3a	77.7a
T_2_	0.41b	0.65b	0.23b	0.26ab	61.5b	68.9b
T_3_	0.39b	0.64b	0.23b	0.25b	62.5b	68.0b
T_4_	0.43a	0.69a	0.24a	0.26ab	61.0b	61.1c
T_5_	0.40b	0.63b	0.23b	0.25b	62.3b	70.1b
T_6_	0.44a	0.70a	0.26a	0.27a	61.4b	62.6c
	0.38a	0.66a	0.23a	0.23b	61.7b	68.0b
Late		0.35c	0.12b			
T_1_	0.23c			0.14b	67.2a	78.9a
T_2_	0.38b	0.60b	0.23a	0.30a	65.0b	73.2b
T_3_	0.38b	0.60b	0.22a	0.28a	64.3b	68.5bc
T_4_	0.41a	0.64a	0.23a	0.29a	62.7b	66.0c
T_5_	0.39b	0.62b	0.23a	0.28a	63.4b	73.1c
T_6_	0.42a	0.63a	0.21a	0.29a	63.0b	67.3c
Average	0.37a	0.61b	0.22a	0.25a	64.2a	71.1a
ANOVA						
Treatments	**	**	*	*	*	*
Seasons	ns	*	ns	*	*	**
S ×T	ns	ns	ns	ns	ns	ns

Note: The treatments coding details already showed in the above table; CF—chemical fertilizer, CM—cattle manure, PM—poultry manure, Ant—anthesis, Mat—physiological maturity, Pos-A—post-anthesis accumulation, NT—nitrogen translocation, NTE—nitrogen translocation efficiency, CNRG—contribution to grain N, NA—Nitrogen accumulation. The mean comparison in columns was made using Tukey tests for treatments mean in both seasons and the lettering was done on basis of Tuky HSD test at 5% using simple effect. ^ns^ = non-significant

*, ** = significant at 5% and ** at 1%, respectively.

The N treated plants showed notably superior post-anthesis N translocation (NT) relative to T_1_ during both seasons, as shown in Table 5. Maximum post-anthesis NT values of 0.27 and 0.29 g hill^-1^ were noted in T_6_ in the early and late seasons, respectively. However, the treatments T_2_, T_3_, T_4_, and T_5_ were statistically non-significant with T_6_. The non-N treated plants showed considerably higher values of 64.3% and 67.2% of N translocation efficiency (NTE), during the early and late season, respectively (Table 5). However, all other N-applied pots were statistically similar (*p* < 0.05) to each other during both seasons. In-addition, higher N translocation contributed to grain (CNRG) was noted in T_1_ (77.7% and 78.9%) in early and late seasons, respectively (Table 5). The treatments T_5_, T_3_, and T_2_ provided the second-highest level of CNRG and were similar to each other. In-contrast, the treatments T_4_ and T_6_ were comparable, but the lowest of all other treatments.

### 3.7. Crop growth, yield, and yield components

Rice growth, yield, and yield components were significantly affected by joint organic and inorganic N treatments and season, but the interaction of treatments and seasons was not non-significant (Table 6). Relative to T_2_, T_6_ increased days to maturity, panicle number, panicle length, and grain yield by 5.5% and 7.2%, 14% and 17%, 16% and 19%, 11% and 16% in the early and late seasons, respectively. However, treatment T_6_ was statistically (p < 0.05) comparable to T_4_. Further, T_3_ and T_5_ also had improved yield and yield components. The lowest yield and yield attributes were noted in T_1_ during both seasons.

**Table 6:** Rice grain yield and yield components under combined organic and inorganic N fertilization.

Treatment	DM	PN	PL	SSP	FGP	TGW	GY
	(days)	(hill^-1^)	(cm)	(panicle^-1^)	(%)	(g)	(g hill^-1^)
Early season	107c		22.9d		79.7c	19.4d	21.1c
T_1_		7.2c		142.2d			
T_2_	108b	9.6b	23.1c	148.1c	85.5a	25.5a	38.6b
T_3_	113ab	9.8b	23.9b	152.3b	84.3b	24.7bc	38.1b
T_4_	114a	11.8a	26.8a	155.5a	85.9a	24.8ab	42.4a
T_5_	113ab	9.9b	23.8b	152.7b	84.1b	23.8b	38.9b
T_6_	114a	11.7a	27.0a	156.1a	85.5a	25.2a	42.8a
Average	111.5b	10.3a	25.4a	151.4a	84.1a	23.9	36.9a
Late season	110c		22.8d		77.5c	19.2d	20.1c
T_1_		7.1c		142.2d			
T_2_	110c	8.9b	22.4c	147.9c	81.3a	24.4a	35.8b
T_3_	115ab	9.4b	23.6b	153.0b	84.9a	22.8b	36.6b
T_4_	116a	10.2a	25.9a	154.5a	85.1a	24.1a	41.1a
T_5_	114b	9.3b	23.5b	152.6b	84.0a	22.8b	36.2b
T_6_	116a	10.3a	26.6a	156.2a	85.2a	24.7a	41.2a
Average	113.5a	9.2b	24.6a	151.1a	80.1b	23.0b	35.1b
ANOVA							
Treatments	**	**	**	**	**	**	**
Seasons	*	*	ns	Ns	*	*	*
S × T	ns	ns	ns	Ns	ns	ns	ns

Note: The treatment coding details already showed in the above table. DM—day to maturity, PN—panicle number, PL—panicle length, SSP, spikelet per panicle, FGP—filled grain percent, TGW—thousand-grain weight, GY—grain yield. The mean comparison in columns was made using Tukey tests for treatments mean in both seasons and the lettering was done on basis of Tukey HSD test at 5% using simple effect. ^ns^ = non-significant

*, ** = significant at 5% and ** at 1%, respectively.

### 3.8. Relation of N accumulation with yield attributes

Variations in the yield attributes greatly depend on N recovery and efficiency. The linear regression analysis demonstrated a very strong and positive correlation between grain yield with panicle number (R^2^ = 0.68* Fig 6A) and spikelets panicle^-1^ (R^2^ = 0.94** Fig 6B). Additionally, linear regression indicated that the N uptake had positively increased the panicle number (R^2^ = 0.78** Fig 6C), and spikelets panicle^-1^ (R^2^ = 0.94** Fig 6D). Therefore, superior N uptake increased the formation of a larger sink (SPP) and thus contributed to superior rice yields.

### 3.9. Pre-and post-anthesis dry matter and N accumulation and translocation and their relationship to grain yield

**Fig 7:**
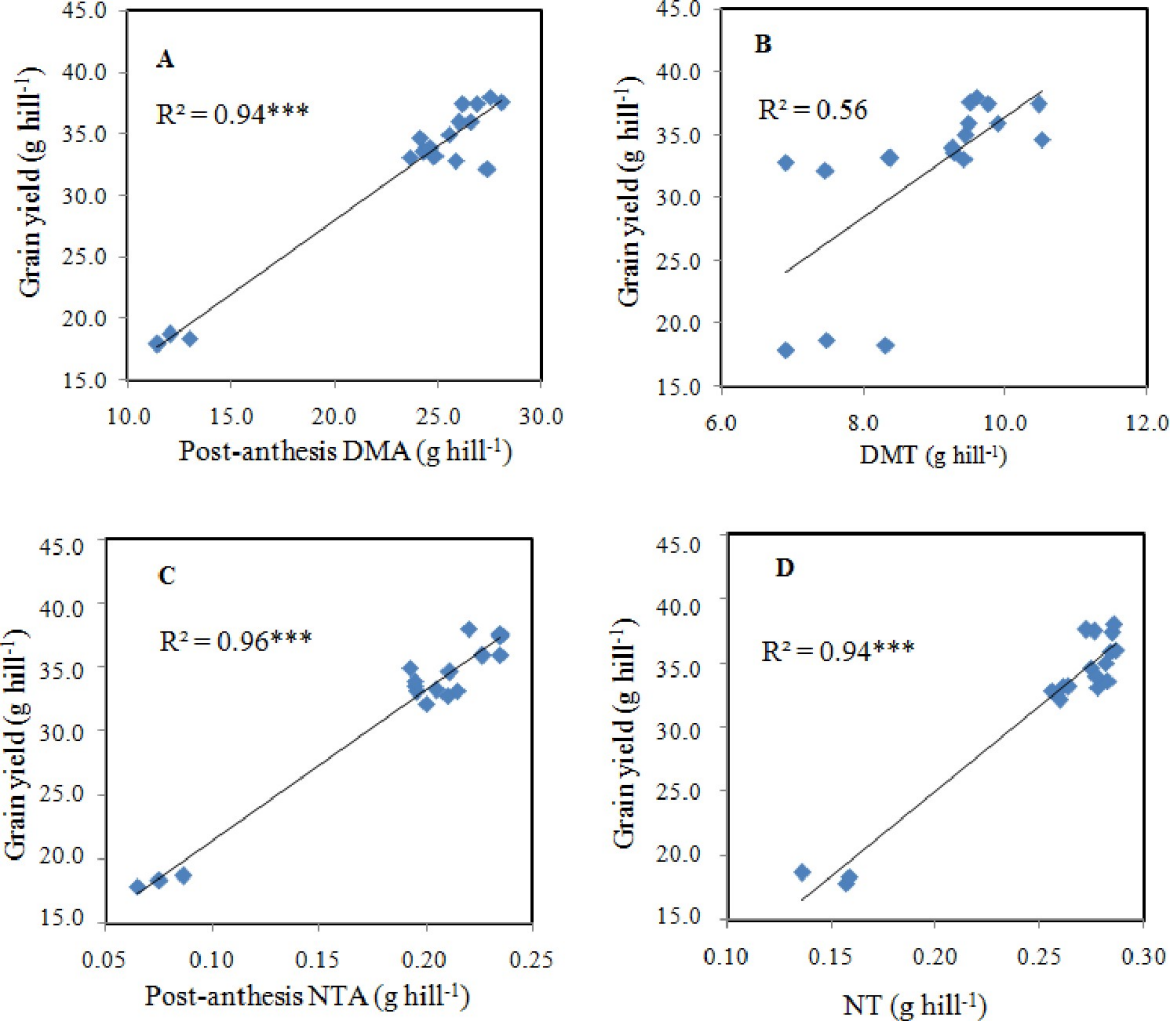
The linear relationship of grain yield with post-anthesis DMA (A), DMA (B), post-anthesis NA (C), and NT (D). *** *p* < 0.0001. Note: DMA-dry matter accumulation, DMT-dry matter translocation, NA-nitrogen accumulation, and NT-N translocation.

**Fig 8:**
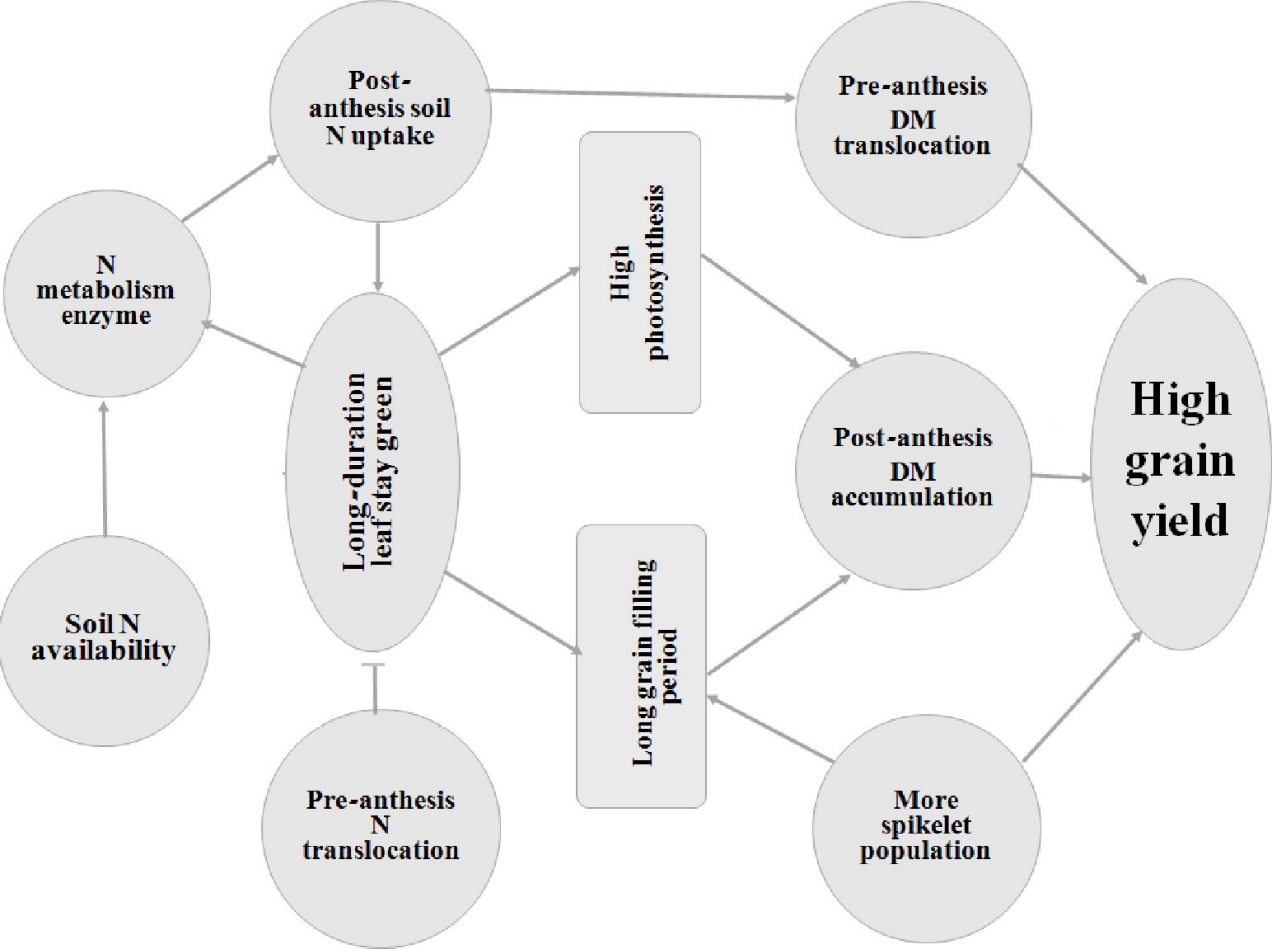
A descriptive model of the role of post-anthesis nitrogen uptake and translocation in grain yield. Arrow “→” indicates induction or promotion, while bar “┬” indicates inhibition.

Increases in cereal grain yields depend on DM and N accumulation and translocation. Our linear regression analysis displayed a highly positive correlation between post-anthesis DM accumulation (R^2^ = 0.95** Fig 7A) and N accumulation (R^2^ = 0.94** Fig 7C) with rice grain yield. In-addition, DM translocation (Fig 8B, R^2^ = 0.58*), and N translocation (Fig 7D R^2^ = 0.93**) accumulated before anthesis also showed a good relationship to rice grain yield. However, the results showed that after anthesis dry matter production was extremely positively associated with rice yield.

## 4. Discussion

The conventional farming system greatly depends on synthetic fertilizer which harmfully affects soil health, the environment, and crop production [46–48]. In order to improve soil health, nutrient status, and sustainable crop production, sustaining soil C and N have recently become key research topics [22, 49]. SOC is a key indicator of soil quality and health [50]. Soil properties are strongly associated with soil organic matter and thus influence soil richness and microbes [51]. Moreover, the accumulation of SOC and its associated environmental importance have strongly improved the ecological activity of soil [52]. Thus, the objective of this study was to determine the effect of a combined application of organic and inorganic fertilizer on rice growth, physiology, yield, and soil properties.

### 4.1. Influence of combined manure and synthetic fertilization on soil quality attributes

In the current study, the concentration of soil TN, SOC, and SOM in the upper layer was considerably enhanced with combined fertilization (Table 3). A substantial improvement in the TN and SOC observed in this study may be linked with the decisive effect of organic fertilizer on the soil C and N change rate, resulting from direct carbon and nitrogen input from organic fertilizer and indirectly through the improvement in crop biomass yields, e.g., root and plant residues [51, 53]. Moreover, the SOC of any specific location depends mainly on the regular returning and recycling of organic materials in the soil [54]. Our findings are in agreement with Purakaiyastha et al. [53], who stated that joint organic manure with chemical fertilizer increased SOC by 11%–80% and TN by 56%–90% in topsoil. Additionally, manure in combination with mineral fertilizer significantly improved the nutrient status of soil (Table 3), tested after harvest in both seasons in the present work. This enhancement in soil nutrients (NPK) was associated with organic manure (cattle or poultry) absorbing more leachate generated during the process, which resulted in enhanced water holding capacity, reduced nutrient leaching, and consequentially more available N, P, and K [51, 55].

The integrated treatments considerably reduced the soil BD in the present study (Table 3). The possible explanation might be the facilitation of root proliferation and its breakdown of the roots, which increased the SOC and led to an increase in soil porosity [52]. The second possible mechanism might be the bulky nature of the manure, which prevented the soil from separating [53], and thus reduced the soil BD. It was also reported that the application of organic fertilizer causes good soil aeration and improvement in soil aggregation, leading to a decrease in soil BD [16, 56]. A similar finding was stated by Mahmood et al [57], who reported organic manure application decreased soil BD, and enhanced soil porosity and water holding capacity.

### 4.2. Leaf photosynthetic and Chl content

Chlorophyll content of leaves has been used to measure plant photosynthetic health [58]. Photosynthesis is the main driver of crop production by enhancing plant growth and biomass production as well as showed a strong response to water and N-supply and uptake [58]. Further, protein and Chl synthesis are also associated with N content of the leaf, as superior *Pn* led to greater stem elongation, leaf area expansion, and delayed leaf senescence [56]. Leaf Chl content and *Pn* are directly affiliated with the N uptake of plants [58]. In this work, leaf *Pn* and chlorophyll contents were found higher in combined organic and inorganic treatments during grain formation relative to the sole urea application treatment (Figs 2 and 3). This rise may be because the combination of manure and chemical fertilizers increases soil health, which decreases the leaching of inorganic elements from the topsoil layer and improves the soil structure and absorption of plant nutrients [59]. Moreover, sufficient water and N supply decrease water-soluble nutrients, and stress-producing root-sourced signal (ABA) leads to stomatal opening and improved leaf water potential and physical activity in leaves [60]. From the present results, we demonstrated that the combined manure and mineral fertilizer treatment improved soil fertility and root growth (Table 3), which ultimately boosted the root’s ability to absorb more water and nutrients, leading to enhanced stomatal conductance, which enhanced the leaf gas exchange attributes and CO_2_ fixing before the heading and milking stages. In-addition, higher *Pn* and Chl content during the grain-filling period in the combined treatment may be due to quicker release of nutrient from synthetic fertilizer and slow and regular release of plant required nutrients from organic manure through the growing period [61].

### 4.3. N-uptake and use efficiencies

In the current farming system, low N use efficiency (NUE) and high N inputs result in high N losses to the environment, causing serious environmental pollution [16, 61]. On the other hand, the organic manure application showed a great increase in NUEs in rice [52, 61]. In the present study, it was observed, that sole urea treated treatment (T_2_) had low AEN (15.6 g g^-1^) and RE_N_ (38%), and combined manure and inorganic fertilizer treated treatment (T_6_) had high AE_N_ (21.2 g g^-1^) and RE_N_ (44%) as shown in Fig 5. This improvement in N use efficiencies under the combined treatments might be due to the improved soil quality in terms of more C and N preservation in soil compared to sole synthetic fertilizer application. Similar to our study, Mehasen et al. [62] concluded that the co-applicatin of manure and synthetic fertilizer improved soil fertility and enhanced nutrients uptake and plant growth. Huang et al. [63] reported that the yield and NUE are much more strongly associated with soil C and TN. In-addition, the application of manure had increased the ability to conserve soil nutrients, eliminated the down side movement of minerals, and hence reduced N leaching [64, 65]. Similarly, other authors reported that organic manure combined with N fertilizer may balance the crop nutrients supply and demand, and improve plant nutrients assimilation and NUE [65, 66]. Several primary enzymes play a significant role in plant N uptake and accumulation [67]. In the present study, the activity of N-metabolizing enzymes, such as NR, GS, and GOGAT was noted higher in integrated treatments (Fig 4). Our findings were similar to those of Sun et al. [67] who stated that the GS and GOGAT activities in functioning flag leaves in the repining period were strongly associated with N accumulation and grain yield of rice. Further, Ceusters et al. [68] also found a close relationship between soil nitrogen accessibility and enhanced N accumulation with the activity of the main assimilatory enzymes of N (NR, GS, and GOGT).

### 4.4. Yield and yield components

The combined application of manure with synthetic fertilizer significantly increased rice growth, yield, and yield components of rice in the present experiment, as shown in Table 6. Compared with control, more productive tillers, longer panicles, and maximum filled grain percentage and grain yield were noted in coupled organic and mineral fertilizer treatment in this study. The improvements in growth and yield components of rice were mainly due to the improved soil fertility under combined treatment in this study (Table 3), which ultimately improved root growth, nutrient uptake, and leaf photosynthetic capacity by providing sufficient macro-and micronutrients from manure and chemical fertilizer throughout the growth period. Our results are also in line with those of Mangalassery et al. [69], who pointed out that the use of manure integrated with chemical fertilizer increased the growth and yield of rice significantly compared to the sole use of chemical fertilizer.

### 4.5. Relation of N recovery to yield attributes

Crop yields strongly relay on yield components, such as PL, PN, and SPP [66]. In this study, our results indicated that both SSP and PN had greatly influenced the grain yield of rice (Table 6). The linear regression analysis confirmed that grain yield was strongly associated with panicle number (R^2^ = 0.67* Fig 6A) and spikelet number (R^2^ = 0.93** Fig 6B). SPP is the main grain yield accumulating factor because the number of increased SPP is directly related to an increase in grain yield [66]. Furthermore, the present study also indicated that PN (R^2^ = 0.78**) and SPP (R^2^ = 0.94**), as shown in Fig 6C and 6D was highly positively correlated with N uptake. Therefore, a higher N uptake and recovery efficiency directly increased the formation of a superior sink (SPP) and ultimately led to maximum grain yield. It is well reported that enough N availability, especially during the grain filling period has enhances spikelet numbers [70]. Hence, the maximum grain yield in this study under the combined treatments is based on sufficient and regular nutrients supply from manure through out the growing season and improved soil fertility, i.e., SOC and TN and reduced BD (Fig 2).

### 4.6. Relationship of DM and N accumulation to grain yield

Biomass production indicates the growth and metabolic potential of plants that regulate the economic output of cereal crops. In this study, the joint treatments (T_6_ and T_4_) had higher values of DMT and DM accumulation after anthesis (Table 5). Further, the linear regression analysis confirmed that post-anthesis DM production (R^2^ = 0.94**) was strongly associated with the grain yield relative to the pre-anthesis DM (R^2^ = 0.56*) as shown in Fig 7A, 7B. From these results, it was observed that post-anthesis biomass production plays a significant role in regulating higher grain yield. Similarly, post-antheis N accumulation also positively correlated with rice grain yield NA (R^2^ = 0.96**) and NT (R^2^ = 0.94** pre-anthesis) is provided in Fig 7C and 7D.

## 5. Conclusion

In the current study, higher biomass production, N-uptake, and grain yield were noted in the combined treatments relative to the CF-only fertilization. This higher N-uptake, biomass production, and grain yield were due to improved soil fertility (SOC, TN, AN, and BD) and flag leaf physiological traits (*Pn* and Chl) during grain-filling, which further promoted DM production. Moreover, the combined treatments had increased the activity of N-metabolizing enzymes and thus increased rice total biomass yield. The linear regression analysis showed that DM and N accumulation after anthesis were highly positively associated with grain yield. Hence, it was noted that DM and N accumulation play a significant role in obtaining high grain yield of rice. It is suggested that PM or CM combined with CF at a ratio of 30:70 is a better plan for achieving maximum rice yields with improved soil health.

## Supporting information

S1 Data (XLSX)

## Abbreviations

N: Nitrogen
CF: Chemical fertilizer
PM: Poultry manure
CM: Cattle manure
GS: Glutamine synthetase
NR: Nitrate reeducates
TN: Total nitrogen
SOC: Soil organic carbon
BD: Bulk density
NT: Nitrogen translocation
DMT: Dry matter translocation
*Pn*: Photosynthetic rate
DAA: Days after anthesis
